# Taste, smell and food-related nausea and vomiting responses in hyperemesis gravidarum: A case-controlled study

**DOI:** 10.1038/s41598-020-61114-y

**Published:** 2020-03-10

**Authors:** Peng Chiong Tan, Balaraman Kartik, Panjaratnam Thanendran, Rozita Zakaria, Sandar Tin Win, Siti Zawiah Omar

**Affiliations:** 1 Department of Obstetrics and Gynecology, Faculty of Medicine, University of Malaya, Lembah Pantai, Kuala Lumpur, 50603 Malaysia; 2 Health Clinic Putrajaya Precint 18, Pusat Pentadbiran Kerajaan Persekutuan, Putrajaya, 62602 Malaysia

**Keywords:** Health care, Vomiting

## Abstract

A case-controlled study was performed to evaluate taste and smell impairment, nausea or vomiting (NV) response to taste and smell and toleration to food texture, item and cooking method in hyperemesis gravidarum patients (HG) compared to gestation-matched controls from a university hospital and primary care clinic in Malaysia. Taste strips (4 base tastes), sniff sticks (16 selected smells) and a food-related questionnaire were used. 124 participants were recruited. Taste impairment was found in 13%(8/62) vs. 0%(0/62) P = 0.003 and the median for correct smell identification was 5[4–6] vs. 9[7–9] P < 0.001 in HG vs. controls. In HG, bitter was most likely (32%) and sweet taste least likely (5%) to provoke NV. In both arms, fish smell was most likely to provoke NV, 77% vs. 32% P < 0.001 and peppermint smell least likely 10% vs. 0% P = 0.012; NV response was significantly more likely for HG arm in 10/16 smells. In HG, worst and best NV responses to food-texture were pasty 69% and crunchy 26%; food-item, plain rice 71% and apple 16% and cooking-style, deep-frying 71% and steaming 55%. HG demonstrated taste and smell impairment and increased NV responses to many tastes and smells. Crunchy sweet uncooked food (apple or watermelon) maybe best tolerated in HG.

## Introduction

More than two thirds of pregnant women experienced nausea and vomiting (NVP) during early pregnancy with the more severe presentation, hyperemesis gravidarum (HG) affecting about 1.2% of pregnancies^1^. In HG, nausea and vomiting is profound resulting in dehydration and starvation with hospitalization typically needed^2,3^.

In HG, sensitivity to sweet taste and taste threshold levels were significantly lower with a considerable alteration in taste perception^4^. A 3-arm study reported that women affected by HG perfomed worst in taste identification when compared to healthy pregnant women or to healthy non-pregnant women, who performed similarly^5^.

Two thirds of the pregnant women rated their smell sensitivity to be enhanced in pregnancy but these self-ratings were not supported by formal test scores^6^. Smell threshold has been found to be higher in first trimester pregnant women but their discrimination of smell intensity was not different^7^. Women in first trimester of pregnancy compared to non-pregnant controls had similar smell identification ability^8^ but this finding is contradicted by another study which has reported a reduction in smell identification ability in the first trimeter of pregnancy compared to women later in pregnancy and to non-pregnant healthy women^9^. A 3-arm study reported that HG women performed the worst in smell identification, followed by healthy pregnant women then healthy non-pregnant women who performed the best^5^. However, the ability by smell to differentiate safe from potentially harmful compounds does not undergo adaptation during pregnancy^10^. In early pregnancy, smell-induced nausea is independent of subjectively perceived intensity and appears to be due to the cognitive processing of olfactory input^11^.

Dietary advice to women affected by NVP such as avoiding spicy or fatty food and preferring bland, dry, high protein food is typically based on expert opinion^12^. In the 12 months before pregnancy, moderate intake of water and adherence to a healthy diet that includes vegetables and fish are associated with a lower risk of developing HG^13^. On the other hand, high daily intake of total fat (primarily saturated fat) prepregnancy increases the risk of HG^14^. It is suggested that a combination of team support, individualized care, supplements created by the dietitian on the basis of patient preferences, and an adapted documented approach for patients with eating aberrations are important aspects of effective management of HG^15^.

We aim to experimentally evaluate taste and smell identification capablility and tolerance and to obtain questionnaire-based data on tolerated food textures, type and cooking method in HG cases compared to gestational-age matched controls to provide a basis for dietary guidance in HG.

## Materials and Methods

The study was approved by the Medical Ethics Committee of University Malaya Medical Center (date of approval: 26 October 2016; reference number: 2016-99-4244) and registered with the Malaysian National Medical Research Registry (reference number: NMRR-16-2027-32724) prior to enrollment of participants. The study was conducted in University Malaya Medical Center, Kuala Lumpur and a state-funded primary care clinic in Putrajaya, Malaysia. Recruited was from December 9, 2016 to June 10, 2017. The study was conducted in accordance with the Declaration of Helsinki on human experimentation. Over six months of recruitment, a total of 124 participants (62 HG cases and 62 controls) were enrolled into the study.

### Study design

This was a prospective case controlled study using (i) standardized Verkauf Taste Strips (Burghart, Wedel, Germany) consisting of sweet, sour, salty and bitter of the same concentration levels, (ii) Verkauf Sniffin’ Sticks (Burghart, Wedel, Germany) of 16 selected smells (banana, honey, lemon, coffee, chocolate, coconut, peppermint, sesame oil, soy sauce, menthol, clove, smoked meat, vinegar, ginger, garlic and fish) and an ad hoc food related questionnaire - Supplementary Fig. S1.

### Participants

Participants were aged ≥18 years with a viable, singleton, intrauterine pregnancy, ≤12 weeks gestation, and enrollment Pregnancy-Unique Quantification of Emesis/Nausea (PUQE) score ≥9 score for HG participants (all were hospitalized for severe nausea and vomiting of pregnancy associated with dehydration and starvation) and ≤6 for controls (all had attended for routine outpatient antenatal care). The severity of NVP can be quantified using the PUQE score^16^. Exclusion criteria were extreme HG symptoms (inability to complete taste and smell tests), language incapacity to respond to questionnaire or pre-existing taste or olfactory disorder.

Eligible women were approached, provided with the patient information sheet and verbally counseled with regard to study participation; written informed consents were obtained from all participants. Participants’ relevant demographic and clinical data were transcribed onto the Case Report Form.

### Study procedures

#### Food questionnaire

At recruitment, participants were asked verbally if they feel more nauseous or feel like vomiting (NV) when considering eating (i) food with texture: crunchy, chewy, soft, pasty and liquid, (ii) specific food items: chicken, white fish, plain white bread, cream crackers plain rice, rice porridge, green vegetables and (iii) fruits: papaya, water melon, pineapple, banana, apple, orange, and grapes, iv) food cooked by: deep-fry, stir-fry, barbeque, steam and roast using a 5-grade Likert scale response - Supplementary Fig. S1.

#### Taste testing

Participants were then tested on their taste identification capability (sweet, sour, salty and bitter) using the disposable “Verkauf Taste Strips”. The strips were placed on the tongue of participants for 5 seconds and up to 30 seconds were allowed for them to identify the taste (without being informed if identification was correct or not) and whether that taste provoked NV, using a 5-grade Likert scale response- Supplementary Fig. S2). Participants rinsed their mouth with plain water, paused for taste effect to dissipate before proceeding to the next taste test.

#### Smell testing

After completing the taste test, smell test was carried out using the 16 different odors of “Verkauf Sniffin’ Sticks” – the selection of these 16 smells by investigators were based on smells familiar to our population. All participants were assessed on the same sequence of smells: banana, honey, lemon, coffee, chocolate, coconut, peppermint, sesame oil, soy sauce, menthol, clove, smoked meat, vinegar, ginger, garlic and fish (sequence based on investigators’ ad hoc ranking of increasing pungence). The odor stick was held 2–3 cm below the nostrils of participants for 5 seconds and participants inhaled to take in the smell. Participants were allowed up to 30 seconds to identify the smell (without being informed if identification was correct or not) and whether that smell provoked NV using a 5-grade Likert scale response- Supplementary Fig. S3. A short pause for smell effect to dissipate was given before proceeding to the next smell test.

### Statistical analyses

Data were entered into a statistical software package SPSS (Version 23, SPSS© Statistics; IBM Corp., Armonk, NY). The t test was used to analyze means and distributions, the Mann-Whitney U test for non-normal data or ordinal data, Fisher exact test for categorical data set if any cell <5 and Chi Square test for larger categorical data sets. All tests were two-sided and p values < 0.05 were reported as significant.

### Ethics approval

Medical Ethics Committee of University Malaya Medical Center (date of approval: 26 October 2016; reference number: 2016-99-4244) and registered with the National Medical Research Registry (reference number: NMRR-16-2027-32724). Written informed consents were taken from all participants.

## Results

Over a six months recruitment period, a total of 124 participants (62 HG cases and 62 controls) were enrolled into the study with no woman approached declining or too unwell (HG cases) to participate. All participants completed the study protocol.

Table 1 depicts the characteristic of study participants stratified according to HG subjects vs. pregnant controls. PUQE score^16^, as expected, were markedly and statistically significantly higher in the HG arm 13 [12–15] vs. 4 [3–5]; P < 0.001. HG women were slightly younger. The remaining characteristics were similar across study arms.

**Table 1:** Characteristics of study participants, stratified to hyperemesis gravidarum cases vs. control.

Characteristics	Hyperemesis (n = 62)	Control (n = 62)	p value
Age (years)	29.5 ± 4.5	31.9 ± 4.6	0.003
Gestational Age (weeks)	8.9 ± 1.8	8.4 ± 1.7	0.17
Ethnicity			0.41
Malay	46 (74)	49 (79)	
Chinese	2 (3)	4 (7)	
Indian	13 (21)	7 (11)	
Others	1 (2)	2 (3)	
Parity			0.11
0	36 (58)	26 (42)	
1	10 (16)	9 (15)	
≥2	16 (26)	27 (43)	
PUQE^a^ Score	13 [12-15] 13.0 ± 1.8	4 [3–5] 4.0 ± 1.8	<0.001

Data expressed as number (%), mean ± standard and median [interquartile range]. Analyses by Student’s t test for continuous data and Chi-square test for larger than 2 × 2 categorical datasets. P < 0.05 for all variables.

^a^Pregnancy-unique quantification of emesis scoring system for nausea and vomiting of pregnancy^16^.

### Food questionnaire

In HG participants, crunchy food texture was reportedly best tolerated with 26% (16/62) stated that it provoked NV, whilst pasty texture had the worst result with 69% (43/62) responded that it provoked NV when they considered eating food with that texture. Among food items, plain rice was worst with NV rate in 71% (44/62) and plain white bread was best at 31% (19/62). Amongst the seven fruits listed, apple fared best with provoked NV rate of only 16% (10/62) and pineapple worst at 57% (35/62). Fruits generally fared well with 5 of the 7 listed occupying the top 5 best tolerated positions amongst the 14 selected food items. Cooked food generally fared poorly in NV provocation rates; steam cooking despite coming in with top tolerance, 55% (34/62) still responded negatively and deep frying had the worst response with 71% (44/62) NV rate amongst HG participants. Controls consistently do not expressed the NV response to any questionnaire items. In every comparison made, HG cases performed statistically significantly worst in NV rates than controls (Table 2).

**Table 2:** Likert scale response of feeling nauseous and vomiting to questionnaire item.

	Hyperemesis (n = 62)		Control (n = 62)		p value
	Agree^a^	Do not Agree^a^	Agree^a^	Do not Agree^a^	
**Food texture**^**a**^					
1. Pasty	43 (69)	19 (31)	0 (0)	62 (100)	<0.001
2. Liquid	29 (47)	33 (53)	0 (0)	62 (100)	<0.001
3. Chewy	24 (39)	38 (61)	0 (0)	62 (100)	<0.001
4. Soft	24 (39)	38 (61)	0 (0)	62 (100)	<0.001
5. Crunchy	16 (26)	46 (74)	0 (0)	62 (100)	<0.001
**Food item**^**b**^					
1. Plain Rice	44 (71)	18 (29)	0 (0)	62 (100)	<0.001
2. Pineapple	35 (57)	27 (43)	0 (0)	62 (100)	<0.001
3. White Fish	34 (55)	28 (45)	0 (0)	62 (100)	<0.001
4. Chicken	32 (52)	30 (48)	0 (0)	62 (100)	<0.001
5. Rice Porridge	26 (42)	26 (58)	0 (0)	62 (100)	<0.001
6. Papaya	26 (42)	36 (58)	0 (0)	62 (100)	<0.001
7. Cream Crackers	25 (40)	37 (60)	0 (0)	62 (100)	<0.001
8. Green Vegetables	25 (40)	37 (60)	0 (0)	62 (100)	<0.001
9. Plain White Bread	19 (31)	43 (69)	0 (0)	62 (100)	<0.001
10. Grapes	19 (31)	43 (69)	0 (0)	62 (100)	<0.001
11. Banana	17 (27)	45 (73)	0 (0)	62 (100)	<0.001
12. Oranges	14 (23)	48 (77)	0 (0)	62 (100)	<0.001
13. Watermelon	13 (21)	49 (79)	0 (0)	62 (100)	<0.001
14. Apples	10 (16)	52 (84)	0 (0)	62 (100)	0.001
**Cooking method**^**c**^					
Deep Fried	44 (71)	18 (29)	0 (0)	62 (100)	<0.001
Barbeque	41(66)	21 (34)	0 (0)	62 (100)	<0.001
Roasted	38 (61)	24 (39)	0 (0)	62 (100)	<0.001
Stir Fried	36 (58)	26 (42)	0 (0)	62 (100)	<0.001
Steamed	34 (55)	28 (45)	0 (0)	62 (100)	<0.001

Data displayed as number (%), analysis by Chi Square test.

^a^Recategorisation of Likert scale responses: Agree includes strongly or somewhat agree, the responses: Do not agree includes neither agree nor disagree, somewhat disagree and strongly disagree responses.

^b^The food texture order as tabulated is from worst to best response in HG women.

^c^The food and fruit item order as tabulated is from worst to best response in HG women.

^d^The cooking method order as tabulated is from worst to best response in HG women.

### Taste tests

In the taste tests (Table 3), controls could invariably (62/62: 100%) identify the basic tastes of sweet, sour, salty and bitter. Controls also invariably (62/62: 100%) did not respond with NV after any taste exposures. HG women were significantly worse than controls in identifying salty taste 90% (56/62) vs. 100% (62/62) P = 0.03. Although a few (2 to 4) HG women were not able to correctly identify taste in a given category, the difference was not significantly at the 5% level for sweet, sour and bitter tastes for HG case-control comparisons. There was taste impairment (at least 1 taste test error in 4) in 14% (9/62) of HG women vs 0% (0/62) of controls P = 0.003. In HG patients, bitter taste was most likely 32% (20/62), followed by sour 23%(14/61) then salty 16% (10/62) and lastly sweet taste the least likely 5% (3/62) to provoke NV response during the taste test (Table 3). From a maximum score of 4, the HG women’s cumulative score (mean ± standard deviation) was 3.76 ± 0.67 compared to 4 ± 0.0, P = 0.006 for controls in number of tastes correctly identified. The NV response rate were significantly higher in the HG arm for bitter, sour and salty but not for sweet when compared to controls.

**Table 3:** Taste identification capability (using Verkauf Taste Test Strips) and nausea-vomiting response to evaluated taste in hyperemesis gravidarum cases vs. controls.

Identification of taste using Verkauf Taste Test Strips						
Taste^a^	Hyperemesis (n = 62)		Control (n = 62)			p value
	Correct	Incorrect	Correct	Incorrect		
Sweet	59 (95)	3 (5)	62 (100)	0 (0)		0.244
Sour	60 (97)	2 (3)	62 (100)	0 (0)		0.496
Salty	56 (90)	6 (10)	62 (100)	0 (0)		0.028
Bitter	58 (94)	4 (7)	62 (100)	0 (0)		0.119
**Likert scale response of feeling nauseous & vomiting to evaluated taste**						
**Taste**^**b**^	**Agree**^**c**^	**Do not Agree**^**c**^	**Agree**^**c**^	**Do not Agree**^**c**^		
Bitter	20 (32)	42 (68)	62 (100)	0 (0)		<0.001
Sour	14 (23)	48 (77)	62 (100)	0 (0)		0.0005
Salty	10 (16)	52 (84)	62 (100)	0 (0)		0.001
Sweet	3 (5)	59 (95)	62 (100)	0 (0)		0.244
**Composite score of taste capability of hyperemesis gravidarum cases vs. controls**						
	**Correct identification of the four tastes**^**d**^ **evaluated**					
**Group**	**None**	**1/4**	**2/4**	**3/4**	**All 4**	
Hyperemesis n = 62	0 (0)	2 (3)	2 (3)	5 (8)	53 (86)	0.003
Control n = 62	0 (0)	0 (0)	0 (0)	0 (0)	62 (100)	

Data displayed as number (%), analysis by Chi Square test.

^a^The taste order as tabulated is according to the test sequence on participants.

^b^The taste order as tabulated is from worst to best response in HG women.

^c^Recategorisation of Likert scale responses: Agree includes strongly or somewhat agree, the responses: Do not agree includes neither agree nor disagree, somewhat disagree and strongly disagree responses.

^d^Sweet, sour, salty and bitter.

^e^2 × 5 Fisher exact test.

### Smell tests

**Figure 1:**
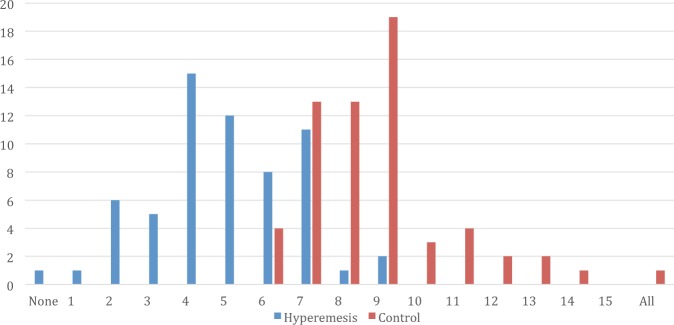
Hyperemesis Gravidarum Compared to Controls: Number of Participants vs. Their Score of Correct Smell Identification. Identification of 16 selected smells (test sequence: banana, honey, lemon, coffee, chocolate, coconut, peppermint, soya sauce, sesame oil, menthol, clove, smoked meat, vinegar, ginger, garlic and fish) The median score [minimum 0 to maximum 16] of smells correctly identified for Hyperemesis Gravidarum group is 5 [IQR 4-6] compared to Control group which is 9 [IQR 7-9] p < 0.001.

In smell identification tests (Table 4) on the 16 smells selected for the study, HG cases performed significantly worse than controls for 10 smells namely coconut, sesame, menthol, chocolate, ginger, peppermint, lemon, garlic, banana and fish compared to controls. From a maximum cumulative score of 16, the HG group scored a median 5 [IQR 4-6] compared to 9 [IQR 7–9] for controls, P < 0.001 in number of smells correctly identified; no woman in the HG scored > 9 correct smell identification (Fig. 1). HG cases compared to controls were more likely to have significantly higher NV response in all 16 smells tested. In HG cases, fish, garlic and sesame oil smells were the most likely to provoke NV responses with rates of 77% (48/62), 63% (39/62) and 58% (36/62) whilst peppermint, lemon and banana smells were best tolerated with rates of 10% (6/62), 15% (9/62) and 15% (9/62) respectively. In controls, fish, smoked meat and garlic smells ranked highest to provoke NV and peppermint, lemon and banana smells rated 0% NV response rate; there was very close symmetry of HG and controls in NV response order to 16 smells tested (Table 4).

**Table 4:** Smell Testing capability (using Verkauf Sniffin Sticks) and nausea-vomiting response to evaluated smell in hyperemesis gravidarum cases vs. controls.

Identification of Selected Common Food smell using Verkauf Sniffing Sticks						
Smell	Hyperemesis (n = 62)		Control (n = 62)		RR (95% CI)	p value
	Incorrect	Correct	Incorrect	Correct		
Fish	20 (32)	42 (68)	0 (0)	62 (100)	—	<0.001
Banana	42 (68)	20 (32)	2 (3)	60 (97)	21.0 (5.3–83.0)	<0.001
Garlic	32 (52)	30 (48)	2 (3)	60 (97)	16.0 (4.0–64.0)	<0.001
Lemon	39 (63)	23 (37)	7 (11)	55 (89)	5.6 (2.7–11.5)	<0.001
Coffee	7 (11)	55 (89)	2 (3)	60 (97)	3.5 (1.0–16.2)	0.16
Peppermint	15 (24)	47 (76)	5 (8)	57 (92)	3.0 (1.2–7.7)	0.02
Ginger	53 (86)	9 (14)	20 (32)	42 (68)	2.7 (1.8–3.9)	<0.001
Chocolate	45 (73)	17 (27)	21 (34)	41 (66)	2.1 (1.5–3.1)	<0.001
Menthol	43 (70)	19 (30)	21 (34)	41 (66)	2.0 (1.4–3.0)	<0.001
Vinegar	51 (82)	11 (18)	44 (71)	18 (29)	1.2 (1.0–1.4)	0.14
Sesame Oil	59 (95)	3 (5)	51 (82)	11 (18)	1.2 (1.0–1.3)	0.04
Coconut	57 (92)	5 (8)	47 (76)	15 (24)	1.2 (1.0–1.4)	0.02
Smoked Meat	60 (97)	2 (3)	56 (90)	6 (10)	1.1 (1.0–1.2)	0.27
Clove	52 (84)	10 (16)	55 (89)	7 (11)	1.0 (1.0–1.1)	0.43
Soy Sauce	58 (93)	4 (7)	56 (90)	6 (10)	1.0 (1.0–1.2)	0.74
Honey	60 (97)	2 (3)	59 (95)	3 (5)	1.0 (1.0–1.1)	1.0
**Likert scale response of feeling nauseous & vomiting to the evaluated smell**						
	**Hyperemesis (n** = **62)**		**Control (n** = **62)**			
**Smell**	**Agree**^**c**^	**Do not Agree**^**c**^	**Agree**^**c**^	**Do not Agree**^**c**^		
Fish	48 (77)	14 (23)	20 (32)	42 (68)	0.3 (0.2–0.5)	<0.001
Garlic	39 (63)	23 (37)	4 (7)	58 (93)	0.4 (0.3–0.6)	<0.001
Sesame Oil	36 (58)	26 (42)	1 (2)	61 (98)	0.4 (0.3–0.6)	<0.001
Vinegar	32 (52)	30 (48)	0 (0)	62 (100)	0.5 (0.4–0.6)	<0.001
Honey	30 (48)	32 (52)	0 (0)	62 (100)	0.5 (0.4–0.7)	<0.001
Smoked Meat	28 (45)	34 (55)	6 (10)	56 (90)	0.6 (0.5–0.8)	<0.001
Soy Sauce	27 (44)	35 (56)	1 (2)	61 (98)	0.6 (0.5–0.7)	<0.001
Clove	23 (37)	39 (63)	2 (3)	60 (97)	0.7 (0.5–0.8)	<0.001
Chocolate	14 (23)	48 (77)	0 (0)	62 (100)	0.8 (0.7–0.9)	<0.001
Coffee	11 (18)	51 (82)	0 (0)	62(100)	0.8 (0.7–0.9)	0.01
Ginger	10 (16)	52 (84)	0 (0)	62 (100)	0.8 (0.8–0.9)	0.01
Menthol	10 (16)	52 (84)	0 (0)	62 (100)	0.8 (0.8–0.9)	0.01
Coconut	10 (16)	52 (84)	0 (0)	62 (100)	0.8 (0.8–0.9)	0.01
Banana	9 (15)	53 (85)	0 (0)	62 (100)	0.9 (0.8–0.9)	0.02
Lemon	9 (15)	53 (85)	0 (0)	62 (100)	0.8 (0.7–0.9)	0.02
Peppermint	6 (10)	56 (90)	0 (0)	62 (100)	0.9 (0.8–1.0)	0.012

Data displayed as number (%), analysis by Chi Square test and P < 0.05 for all variables.

^a^The smell order as tabulated is from worst to best performance as stratified by relative risk for incorrect smell identification comparing HG with controls.

^b^The smell order as tabulated from worst to response in HG women.

^c^Recategorisation of Likert scale responses: Agree includes strongly or somewhat agree, the responses: Do not agree includes neither agree nor disagree, somewhat disagree and strongly disagree responses.

## Discussion

In our experiment using taste pad and smell stick testing, we find impairment (14% vs. 0%) of taste and significantly lower cumulative (out of 16 smells tested) correct smell identification score 5 [IQR 4–6] vs. 9 [IQR 7-9] in the HG patients compared to early pregnancy controls. Taste identification deficit to sweet, sour, salty and bitter between HG cases and controls were more subtle with 86% of HG cases maintaining intact taste identification capability to all 4 basic tastes compared to the larger smell identification deficit for the 16 selected smells in HG arm.

In a 3-arm study comprising HG affected women, healthy pregnant women and healthy non-pregnant women, with 4 base tastes assessed using taste sprays, taste identification scores were respectively 3.4 ± 0.9 vs. 3.9 ± 0.1 vs. 3.9 ± 0.1 p = 0.003^5^ whereas in our study we used individual taste strips with scores of 3.76 ± 0.67 (HG women) compared to 4 ± 0.0 (early pregnancy controls), P = 0.006: these findings were consistent with each other in demonstrating a small reduction in taste identification ability in HG. With 12 odors assessed using Sniffin’ Sticks test battery (Burghart, Wedel, Germany) as similarly used in our study, smell identification scores were respectively 9.1 ± 1.6 (HG women) vs 9.3 ± 1.4 (healthy pregnant women) vs 10.1 ± 1.3 P = 0.03 (healthy non pregnant women)^5^ compared to our finding of 5 [IQR 4-6] (HG women) vs 9 [IQR 7-9] (early pregnancy control) P < 0.001 of 16 smells tested (there were 6 overlap in the selected smells between their study and ours); our finding showed a much more marked reduction in odor identification ability in HG affected women versus early pregnancy controls compared to the marginal difference (9.1/12 vs 9.3/12) between these 2 groups in their study.

Sweet is the best tolerated taste by the HG group with only 5% finding it disagreeable, the only taste or smell where NV response rate is not significantly different to controls. On the other hand, universally in controls no taste test provoked any NV response. Women with NVP are characterized by high intakes of carbohydrates and added sugar^17^ and according to a multiethnic South African study pregnant women have pronounced craving for sweet foods^18^, our data that HG women find sweet taste least likely to provoke NV is consistent with a consumption pattern that favored added sugar in NVP women and craving for sweet foods in pregnancy.

The smell of garlic and sesame oil for instance had the largest absolute difference of 56% in NV response rates of the 16 smells tested between HG cases to controls. Peppermint is the best tolerated smell in HG women with only 10% reporting NV response after test exposure compared to 0% in controls. There was close symmetry in the smell-provoked NV response league table for HG and controls, with good matches at the top and bottom of the respective rankings but vinegar and honey smells are qualitative standouts; 52% and 48% NV response in HG compared to 0% and 0% NV response in controls (Table 4). It has been reported that pregnant women rated the odors of ‘rum’, ‘cigarette’ and ‘coffee’ as more aversive than non-pregnant women^19^; in our controls coffee smell had 0% provoke NV response rate and in HG coffee had a relatively low 18% NV response rate. Meat, fish, coffee and fatty foods, are foods most often avoided in pregnancy according to a multiethnic South African study^18^, consistent with the high ranked position in our NV response table of fish (77%) and smoked meat odors (45%) for HG women but our controls also ranked these smells (32% and 10% respectively) as NV inducing (Table 4) albeit at a diminished rate. Ginger taken orally has been shown to help nausea and vomiting in pregnancy^20^; ginger smell had a low NV response rate of 16% and 0% for HG and controls respectively.

In the HG group, the magnitude of NV response to many (8 of 16 had 37% or higher NV response) smells were larger than to the 4 base tastes (bitter with worst 32% NV response) suggesting that the smell more than taste of food and drink may have the greater potential to be the initiator and driver of nausea and emesis in HG.

The cause or effect of taste and smell deficits to HG cannot be established by this cross-sectional study; we do not have the longitudinal data to evaluate if these deficits predate or dissipate with recovery from HG.

In the questionnaire-based component of our study, crunchy (26%) food texture is least likely to evoke feel more nauseous or feel like vomiting responses and pasty texture (69%) the most likely. Controls did not have a NV response to any of the five foods texture evaluated.

Of the 14 selected common food items, plain rice had the highest (71%) NV in HG cases compared to 0% in controls. The poor response to plain rice may be due to its ethno-cultural symbolism as “food” in our society and probably the rejection of food conditioned by acute HG. Rice porridge, broadly seen as a recuperative food during illness in our social setting had a mid-ranking position in our food item league table with 42% NV response. Aversions to staple foods is reportedly common in pregnancy^21^ and our finding is consistent with an exaggerated response in HG.

Fruits occupy the best 5 positions (out of 7 fruits listed); apple was in best position with only a 16% NVP response rate in HG cases. Pineapple’s and to a lesser extent papaya’s poor rating amongst fruits might be accounted for by folk belief that they induce miscarriage with that concern amplified by vulnerability arising from HG. None of the 14 food items evaluated evoked NV sensation in controls.

Contemplating eating steamed food had the lowest NV response rate at 55%; deep-fried of food had the highest at 71% in HG. With all five cooking methods, a majority in HG arm expressed a NV response suggesting that the thought of eating cooked food was problematic. None of the five cooking methods evoked NV sensation in controls.

There was a confluence of findings that fruity (lemon, banana and coconut) smells were better tolerated and fruits like apples, watermelon, oranges and bananas least likely to provoke NV sensations when their eating was contemplated. Food texture, cooking method and food item in terms of tolerance or preference were likely influenced by culinary heritage. However, preference for and tolerance to fresh fruits are quite likely to cut across the ethno-cultural and culinary boundaries. The fruits selected for our questionnaire are widely consumed. We believe our finding that generally favored fresh fruits in HG could be generalizable.

In pregnant women in Tanzania, fruits like mangos and oranges are craved and rice and fish avoided usually for no particular reason^22^. Our data for HG women finds oranges to be well tolerated and rice and fish amongst the worst tolerated reflecting a degree of symmetry with the Tanzanian data. Increasing severity of nausea was also associated with decreasing prudent diet score from before to early pregnancy, such that women with severe nausea had prudent diet scores 0.29 SDs lower than those with no nausea (P < 0.001)^23^.

### Strengths and limitations

This original study provided cross sectional data on the association of taste and smell defects in HG compared to controls. The selection of smells for our test panel of 16 is eclectic but commonly encountered in our population. This smells panel may restrict generalizability to other HG populations. Although there is some overlap, our test panel of smell did not dovetail perfectly with the food items in the questionnaire section of our study; possibly a missed opportunity to corroborate across the different sections of this study.

## Conclusion

There is a deficiency in taste and smell identification in women hospitalized for HG. Women affected by HG were also hypersensitive to taste (except sweet taste) and particularly smell stimulation with significantly more women reporting NV when experimentally exposed compared to gestation-matched controls. Sweet, crunchy and uncooked (fresh) food characteristics were favored by HG women, and these characteristics dovetailed neatly to apple and watermelon being rated as best tolerated food when their eating is contemplated.

## Supplementary information

Supplementary information.

## Data Availability

All data generated or analyzed during this study are included in this published article (and its Supplementary information files).
