# Supplementary material for: Taste, smell and food-related nausea and vomiting responses in hyperemesis gravidarum: A case-controlled study
Source: Sci Rep. 2020 Mar 10;10:4445. doi: 10.1038/s41598-020-61114-y (PMC7064589; doi:10.1038/s41598-020-61114-y)
Supplement: Supplementary file 1 — Supplementary information. [file 41598_2020_61114_MOESM1_ESM.pdf]

Taste, smell and food-related nausea and vomiting responses in hyperemesis gravidarum: A case-controlled study

Authors
Peng Chiong Tan
Balaraman Kartik
Panjaratnam Thanendran
Rozita Zakaria
Sandar Tin Win
Siti Zawiah Omar

Supplementary Figure S1. Food Questionnaire

(i) Food texture

I feel more nauseous or feel like vomiting when considering eating food that is

A. CRUNCHY

STRONGLY DISAGREE	DISAGREE	NEITHER DISAGREE OR AGREE	AGREE	STRONGLY AGREE

B. CHEWY

STRONGLY DISAGREE	DISAGREE	NEITHER DISAGREE OR AGREE	AGREE	STRONGLY AGREE

C. SOFT

STRONGLY DISAGREE	DISAGREE	NEITHER DISAGREE OR AGREE	AGREE	STRONGLY AGREE

D. PASTY

STRONGLY DISAGREE	DISAGREE	NEITHER DISAGREE OR AGREE	AGREE	STRONGLY AGREE

E. LIQUID

STRONGLY DISAGREE	DISAGREE	NEITHER DISAGREE OR AGREE	AGREE	STRONGLY AGREE

(ii) Specific food items

I feel more nauseous or feel like vomiting when considering eating food that is

A.CHICKEN

STRONGLY DISAGREE	DISAGREE	NEITHER DISAGREE OR AGREE	AGREE	STRONGLY AGREE

B. WHITE FISH

STRONGLY DISAGREE	DISAGREE	NEITHER DISAGREE OR AGREE	AGREE	STRONGLY AGREE

C. PLAIN WHITE BREAD

STRONGLY DISAGREE	DISAGREE	NEITHER DISAGREE OR AGREE	AGREE	STRONGLY AGREE

D. CREAM CRACKERS

STRONGLY DISAGREE	DISAGREE	NEITHER DISAGREE OR AGREE	AGREE	STRONGLY AGREE

E. PLAIN RICE

STRONGLY DISAGREE	DISAGREE	NEITHER DISAGREE OR AGREE	AGREE	STRONGLY AGREE

F. RICE PORRIDGE

STRONGLY DISAGREE	DISAGREE	NEITHER DISAGREE OR AGREE	AGREE	STRONGLY AGREE

G. GREEN VEGETABLES

STRONGLY DISAGREE	DISAGREE	NEITHER DISAGREE OR AGREE	AGREE	STRONGLY AGREE

(iii) Fruits

• PAPAYA

STRONGLY DISAGREE	DISAGREE	NEITHER DISAGREE OR AGREE	AGREE	STRONGLY AGREE

• WATER MELON

STRONGLY DISAGREE	DISAGREE	NEITHER DISAGREE OR AGREE	AGREE	STRONGLY AGREE

• PINEAPLE

STRONGLY DISAGREE	DISAGREE	NEITHER DISAGREE OR AGREE	AGREE	STRONGLY AGREE

• BANANA

STRONGLY DISAGREE	DISAGREE	NEITHER DISAGREE OR AGREE	AGREE	STRONGLY AGREE

• APPLES

STRONGLY DISAGREE	DISAGREE	NEITHER DISAGREE OR AGREE	AGREE	STRONGLY AGREE

• ORANGES

STRONGLY DISAGREE	DISAGREE	NEITHER DISAGREE OR AGREE	AGREE	STRONGLY AGREE

• GRAPES

STRONGLY DISAGREE	DISAGREE	NEITHER DISAGREE OR AGREE	AGREE	STRONGLY AGREE

(iv) **Cooking methods**

I feel more nauseous or feel like vomiting when considering eating food that is

- DEEP FRIED

STRONGLY DISAGREE	DISAGREE	NEITHER DISAGREE OR AGREE	AGREE	STRONGLY AGREE

- STIR FRIED

STRONGLY DISAGREE	DISAGREE	NEITHER DISAGREE OR AGREE	AGREE	STRONGLY AGREE

- BARBEQUE

STRONGLY DISAGREE	DISAGREE	NEITHER DISAGREE OR AGREE	AGREE	STRONGLY AGREE

- STEAMED

STRONGLY DISAGREE	DISAGREE	NEITHER DISAGREE OR AGREE	AGREE	STRONGLY AGREE

- ROASTED

STRONGLY DISAGREE	DISAGREE	NEITHER DISAGREE OR AGREE	AGREE	STRONGLY AGREE

Supplementary Figure S2. Taste Testing

I feel more nauseous or feel like vomiting when exposed to this taste

- SWEET

STRONGLY DISAGREE	DISAGREE	NEITHER DISAGREE OR AGREE	AGREE	STRONGLY AGREE

IDENTIFY TASTE AS:

- SOUR

STRONGLY DISAGREE	DISAGREE	NEITHER DISAGREE OR AGREE	AGREE	STRONGLY AGREE

IDENTIFY TASTE AS:

- SALTY

STRONGLY DISAGREE	DISAGREE	NEITHER DISAGREE OR AGREE	AGREE	STRONGLY AGREE

IDENTIFY TASTE AS:

- BITTER

STRONGLY DISAGREE	DISAGREE	NEITHER DISAGREE OR AGREE	AGREE	STRONGLY AGREE

IDENTIFY TASTE AS:

Supplementary Figure S3. Smell Testing

I feel more nauseous or feel like vomiting when exposed to this smell

- BANANA

STRONGLY DISAGREE	DISAGREE	NEITHER DISAGREE OR AGREE	AGREE	STRONGLY AGREE

SMELL CORRECTLY IDENTIFIED: YES / NO

- HONEY

STRONGLY DISAGREE	DISAGREE	NEITHER DISAGREE OR AGREE	AGREE	STRONGLY AGREE

SMELL CORRECTLY IDENTIFIED: YES / NO

- LEMON

STRONGLY DISAGREE	DISAGREE	NEITHER DISAGREE OR AGREE	AGREE	STRONGLY AGREE

SMELL CORRECTLY IDENTIFIED: YES / NO

- COFFEE

STRONGLY DISAGREE	DISAGREE	NEITHER DISAGREE OR AGREE	AGREE	STRONGLY AGREE

SMELL CORRECTLY IDENTIFIED: YES / NO

- CHOCOLATE

STRONGLY DISAGREE	DISAGREE	NEITHER DISAGREE OR AGREE	AGREE	STRONGLY AGREE

SMELL CORRECTLY IDENTIFIED: YES / NO

- COCONUT

STRONGLY DISAGREE	DISAGREE	NEITHER DISAGREE OR AGREE	AGREE	STRONGLY AGREE

SMELL CORRECTLY IDENTIFIED: YES / NO

- PEPPERMINT

STRONGLY DISAGREE	DISAGREE	NEITHER DISAGREE OR AGREE	AGREE	STRONGLY AGREE

SMELL CORRECTLY IDENTIFIED: YES / NO

- SESAME OIL

STRONGLY DISAGREE	DISAGREE	NEITHER DISAGREE OR AGREE	AGREE	STRONGLY AGREE

SMELL CORRECTLY IDENTIFIED: YES / NO

- SOY SAUCE

STRONGLY DISAGREE	DISAGREE	NEITHER DISAGREE OR AGREE	AGREE	STRONGLY AGREE

SMELL CORRECTLY IDENTIFIED: YES / NO

- MENTHOL

STRONGLY DISAGREE	DISAGREE	NEITHER DISAGREE OR AGREE	AGREE	STRONGLY AGREE

SMELL CORRECTLY IDENTIFIED: YES / NO

- CLOVE

STRONGLY DISAGREE	DISAGREE	NEITHER DISAGREE OR AGREE	AGREE	STRONGLY AGREE

SMELL CORRECTLY IDENTIFIED: YES / NO

- SMOKED MEAT

STRONGLY DISAGREE	DISAGREE	NEITHER DISAGREE OR AGREE	AGREE	STRONGLY AGREE

SMELL CORRECTLY IDENTIFIED: YES / NO

- VINEGAR

STRONGLY DISAGREE	DISAGREE	NEITHER DISAGREE OR AGREE	AGREE	STRONGLY AGREE

SMELL CORRECTLY IDENTIFIED: YES / NO

- GINGER

STRONGLY DISAGREE	DISAGREE	NEITHER DISAGREE OR AGREE	AGREE	STRONGLY AGREE

SMELL CORRECTLY IDENTIFIED: YES / NO

- GARLIC

STRONGLY DISAGREE	DISAGREE	NEITHER DISAGREE OR AGREE	AGREE	STRONGLY AGREE

SMELL CORRECTLY IDENTIFIED: YES / NO

- FISH

STRONGLY DISAGREE	DISAGREE	NEITHER DISAGREE OR AGREE	AGREE	STRONGLY AGREE

SMELL CORRECTLY IDENTIFIED: YES / NO
